# Influences of the priming procedure and saline circulation conditions on polyvinylpyrrolidone *in vitro* elution from polysulfone membrane dialyzers

**DOI:** 10.1016/j.bbrep.2021.101140

**Published:** 2021-10-06

**Authors:** Yoshinori Sato, Hayato Horiuchi, Shinji Fukasawa, Shingo Takesawa, Jun Hirayama

**Affiliations:** a Department of Clinical Engineering, Faculty of Health Sciences, Komatsu University, Ishikawa, Japan; b Department of Medical Engineering, National Centre for Child Health and Development, Tokyo, Japan; c Department of Medical Engineering, Kyushu University of Health and Welfare, Miyazaki, Japan

**Keywords:** Polysulfone membrane dialyzer, Polyvinylpyrrolidone, Hemodialysis

## Abstract

In hemodialysis (HD), the patient's blood is purified via circulation in an extracorporeal circuit containing a dialyzer. In the manufacturing process of polysulfone (PSu) membrane dialyzers, the membranes are hydrophilized via the addition of the hydrophilic agent polyvinylpyrrolidone (PVP) to increase their hydraulic permeability. The elution of PVP from the membrane reduces the membrane's hydraulic permeability, and the eluted PVP could cause adverse effects in the human body. Therefore, it is important to identify the factors that induce PVP elution from PSu dialyzer membranes to improve the efficiency and safety of HD. In the present study, experimental circuits connecting each of the three types of PSu membrane dialyzers that had been sterilized, using gamma irradiation, autoclaving, or in-line steam methods, were prepared. After the dialyzers were primed, saline was circulated in the circuits at a flow rate of 100 mL/min or 200 mL/min. At 0, 2, 4, 6, and 8 h after circulation was initiated, the amount of PVP eluted from the PSu membranes *in vitro* was determined. In this experimental setting, longer the circulation duration, greater the amount of PVP eluted from the PSu membranes of the tested dialyzers; however, the flow rate did not influence the *in vitro* elution of PVP. Furthermore, the immersion of the dialyzer membranes in saline for 24 h strongly facilitated the *in vitro* elution of PVP. In sum, these results suggest that the duration of PSu membrane incubation in saline is a determinant of the level of PVP elution from the PSu membrane dialyzers.

## Highlights

• The flow rate did not influence the *in vitro* PVP elution from PSu membrane dialyzers.
• The time of PSu membrane incubation in saline is a determinant of PVP elution level.
• Substances other than PVP are eluted from PSu membrane dialyzers.

## Graphical abstract

**Figure:**
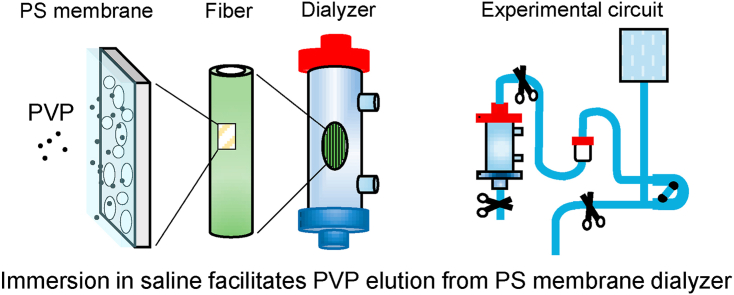

## 1 Introduction

Hemodialysis (HD) is a medical technique wherein a patient's blood is purified by circulating it through an extracorporeal blood circuit containing a dialyzer [1]. HD therapy has been used in the treatment of end-stage kidney diseases for more than half a century [2,3]. Recently, it was applied in a wide range of disease treatments, including continuous renal replacement therapy (CRRT) and prerenal acute renal failure in patients with multiple organ failure [4]. The blood is circulated in the extracorporeal circuit for 3–8 h for the HD therapy of patients with end-stage kidney diseases, whereas the blood circulation duration needs to be much longer, such as 24 h, for CRRT.

Various membrane materials have been developed to improve the solute clearance of the dialyzer and its biocompatibility. Polysulfone (PSu), a synthetic polymer membrane material, possesses high permeability for solutes and excellent biocompatibility, which make it the most clinically used dialyzer membrane material [5,6]. PSu is highly hydrophobic; therefore, the PSu membranes are hydrophilized by the addition of polyvinylpyrrolidone (PVP), a hydrophilic agent, to increase their hydraulic permeability [[7], [8], [9]]. It is noteworthy that the induction of adverse effects, such as thrombocytopenia, blood pressure reduction, and abnormal production of antibodies, in the patients with HD therapy using PSu membrane dialyzers has been reported [[10], [11], [12], [13], [14], [15], [16]]. In addition, subchronic toxicity effects of PVP exposure and intake in organs and tissues are well recognized [17]. Accordingly, minimization of the PVP elution from the dialyzer membrane during HD is necessary to avoid the reduction in the hydraulic permeability of membrane and PVP-induced adverse effects.

Survival is reported to be significantly higher in patients undergoing dialysis with ≥4 h of blood circulation in a single session than that in patients with <4 h circulation in a single session [[18], [19], [20], [21]]. Furthermore, it has been reported that the risk of 1-year patient mortality was reduced with a dialysis blood flow rate >200 mL/min in comparison with that of 200 mL/min [21]. These findings suggest that a longer duration of blood circulation per session and a higher blood flow rate improve the prognosis of dialysis patients. In contrast, the effects of the duration of circulation and the dialysate flow rate in a dialysis session on the elution of PVP from the dialyzer membrane remain to be examined.

One of the essential processes for the use of dialyzers in HD therapy is their sterilization, which is performed using several methods, such as gamma irradiation, autoclaving, and in-line steam methods [22,23]. Thereafter, the sterilized dialyzers are required to undergo priming process before they are used for circulating the patient's blood. In the priming process, the dialyzers are washed with a washing solution, such as saline, to remove undesired materials; then, the dialyzers are filled with the dialysis fluid.

In the present study, we evaluated the influences of the priming process (washing with saline), dialysis circulation conditions, and saline incubation on PVP elution from each of three types of PSu membrane dialyzers sterilized using gamma irradiation, autoclaving, or in-line steam methods.

## 2 Materials and methods

### 2.1 Dialyzers

In this study, three types of PSu membrane dialyzers and one non-PSu membrane dialyzer were used. Their characteristics have been summarized in Table 1. The PSu membrane dialyzers were sterilized using gamma irradiation, autoclaving, or in-line steam methods, and are described as PSu dialyzer (Gamma IR), PSu dialyzer (Autoclave), and PSu dialyzer (In-line steam), respectively, hereafter. The non-PSu membrane dialyzer is a cellulose triacetate (CTA) membrane dialyzer.

**Table 1:** Characteristics of dialyzers used.

Description in the current study	Product name (Maker)	Membrane material	Sterilization method	Surface area (m^2^)
Polyvinylpyrrolidone (PSu) membrane dialyzer (Gamma IR)	NV-15X (Toray Industries, Inc.)	PSu	Gamma irradiation	1.5
PSu membrane dialyzer (Autoclave)	RENAK-PS1.6 (Kawasumi Laboratories, Inc.)	PSu	Autoclave	1.6
PSu membrane dialyzer (In-line stem)	FX-140J (Fresenius Medical Care)	PSu	In-line steam	1.4
Cellulose triacetate (CTA) membrane dialyzer	FB-150U (NIPRO)	CTA	Gamma irradiation	1.5

### 2.2 Preparation of the experimental circuits

**Fig. 1:**
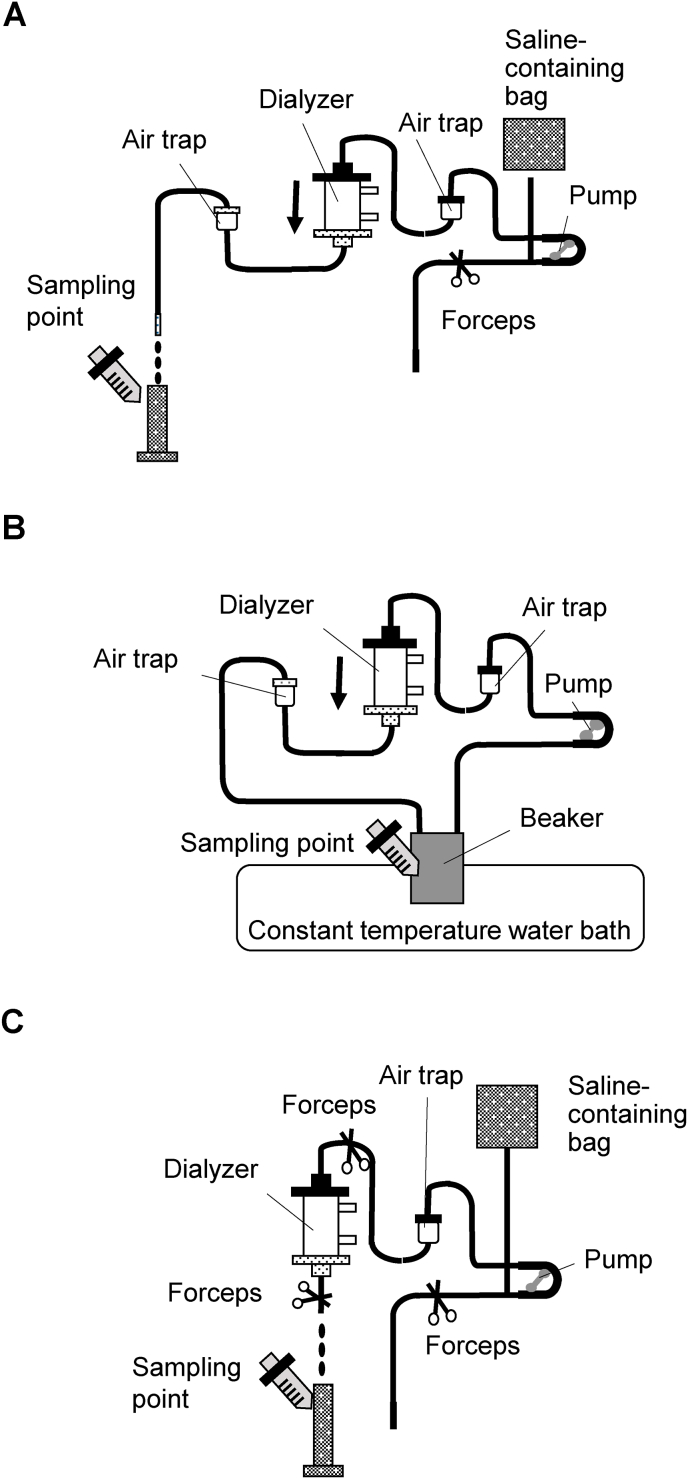
Experimental circuits. Schematic diagrams of the experimental circuits used to evaluate the influences of priming (A), circulation of saline (B), and incubation with saline (C) on polyvinylpyrrolidone (PVP) elution from the polysulfone membrane dialyzers. The dialysis fluid ports of the dialyzer were clamped when perfusing the filter. The arrows in A and B illustrate the perfusion direction. The pictures of syringes indicate the points from which the analytical samples for the measurement of the amount of PVP were collected.

To test the effects of the priming process on the PVP *in vitro* elution caused by the PSu and non-PSu dialyzer membranes, the experimental circuit illustrated in Fig. 1A was connected to the blood pump of the dialysis machine. Then, 1000 mL of saline (0.9% NaCl) was made to flow in the circuit at a flow rate of 100 mL/min, and the saline was collected from the circuit exit in 250-mL samples for determining the PVP content.

To examine the effects of the circulation conditions on the PVP *in vitro* elution from the dialyzer membranes, the experimental circuit illustrated in Fig. 1B was built. The dialyzers in the circuit were washed with circulating 1000 mL of saline throughout the circuit. Thereafter, 500 mL of fresh saline (37 °C) was poured into the beaker in the thermostatic bath and circulated in the experimental circuit at a flow rate of either 100 mL/min or 200 mL/min. The analytical samples for measuring the amount of PVP were collected from the beaker at 0, 2, 4, 6, and 8 h after circulation was started.

To examine the effect of incubation of the dialyzer membranes with saline on the PVP elution from the primed dialyzers, the experimental circuit illustrated in Fig. 1C was built. The circuit was filled with saline and thereafter incubated at 25 °C for 24 h. After the incubation, the saline was recovered from the circuit for measuring the PVP amount.

### 2.3 Measurement of the PVP amount

The PVP concentration was measured using the Müller method. As per this method, the PVP concentration was determined using a spectrophotometer (Hitachi; U-5100) to measure the absorbance from the formation of PVP–iodine complexes after the addition of iodine. PVP (K90) was used to create the standard curve for measurement of the PVP amount.

### 2.4 Statistical analyses

The statistical significance was calculated using Statcel4 software (OMS Publisher). First, the normality of the distribution was evaluated by comparing the results from the experiments with the four different dialyzers. Normality was not confirmed; thereafter, multigroup analysis was performed using the Steel–Dwass method. The statistical analysis of the two groups of 100 mL/min and 200 mL/min flow rates was performed using the Mann–Whitney's *U* test.

## 3 Results and discussion

### 3.1 PVP *in vitro* elution levels from each of the PSu membrane dialyzers using the priming treatment

**Fig. 2:**
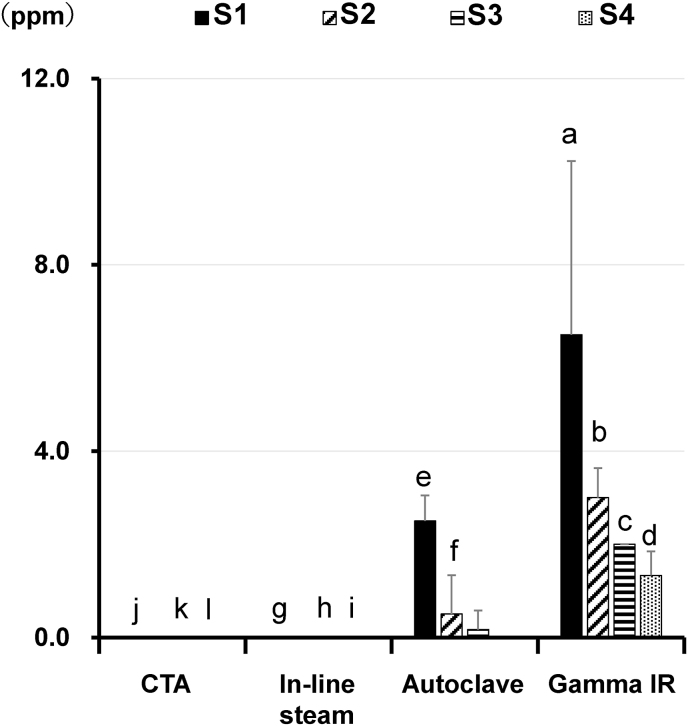
Profiles of polyvinylpyrrolidone *in vitro* elution obtained from the dialyzers by washing them with saline. Each dialyzer was set in the circuit as described in Fig. 1A. Thereafter, 1000 mL of saline was flowed in the experimental circuit at a flow rate of 100 mL/min and was collected from the circuit exit in 250-mL samples for measuring the polyvinylpyrrolidone (PVP) concentration using spectrophotometry. The analytical samples were named S1, S2, S3, and S4 in the order of their collection. Values represent the mean ± standard error of the six independent experiments. P < 0.01: a vs. e; a vs. g; a vs. k. P < 0.05: b vs. f; c vs. h; c vs. k; d vs. i; d vs. l. Abbreviations: Gamma IR; polysulfone dialyzer (Gamma irradiation), Autoclave; polysulfone dialyzer (Autoclave), In-line steam; polysulfone dialyzer (In-line steam), CTA; cellulose triacetate membrane dialyzer.

In the clinical priming process, the dialyzers are washed with a washing solution, such as saline [24]. To examine the influence of saline washing on the PVP *in vitro* elution from PSu and non-PSu membranes, the experimental circuit described in Fig. 1A was set up and connected to the blood pump of a dialysis machine. Then, 1000 mL of saline was made to flow in the circuit at a flow rate of 100 mL/min, and we collected 250-mL samples of saline for analysis from the circuit exit. The samples were named S1, S2, S3, and S4 in the order of their collection. As shown in Fig. 2, washing with saline induced PVP elution from the PSu dialyzer (Gamma IR). The amount of PVP eluted decreased based on the order of the sample collection. A similar decrease with sample collection was noted for the PSu dialyzer (Autoclave). In contrast to these results, we did not detect PVP elution from the dialyzer (In-line steam). It is noteworthy that no PVP elution was detected from the CTA membrane dialyzer (non-PSu membrane dialyzer) that did not contain PVP, confirming the specificity of the PVP measurement system used in the present study. In this experiment, whether all PVP *in vitro* elution from the membrane for each dialyzer was induced by washing with 1000 mL saline could not be concluded. Thus, we conducted the following experiments using dialyzers that had been washed with 1000 mL of saline (primed dialyzers).

### 3.2 Evaluation of the effects of the circulating conditions on PVP *in vitro* elution from the primed dialyzers

**Fig. 3:**
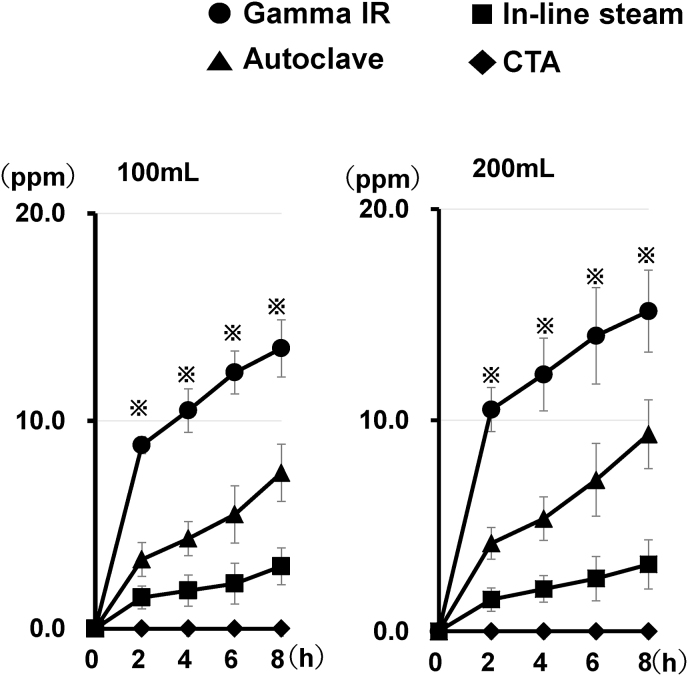
Effects of the circulation duration and flow rate of the saline on polyvinylpyrrolidone *in vitro* elution from the primed dialyzers. Each primed dialyzer was set in the circuit as described in Fig. 1B. Saline was then flowed in the experimental circuit at a flow rate of 100 mL/min (left panel) or 200 mL/min (right panel). At the indicated time points after the start of the circulation, polyvinylpyrrolidone (PVP) concentration in the beaker was determined. Values represent the mean ± standard error of the six independent experiments. ^**※**^P < 0.05. Abbreviations: Gamma IR; polysulfone dialyzer (Gamma irradiation), Autoclave; polysulfone dialyzer (Autoclave), In-line steam; polysulfone dialyzer (In-line steam), CTA; cellulose triacetate membrane dialyzer.

We examined effects of the circulation duration on PVP *in vitro* elution from each primed dialyzer. First, the saline was circulated in the experimental circuit at a flow rate of 100 mL/min. Thereafter, the samples for analysis were collected at 0, 2, 4, 6, and 8 h after the start of the circulation. In the experiment with the PSu dialyzer (Gamma IR), elution of PVP from the dialyzer's membrane was detected 2 h after circulation was started and reached high levels 8 h after the circulation started (Fig. 3; Left). Similar patterns were observed for PVP elution from the PSu dialyzer (Autoclave) and PSu dialyzer (In-line steam). No PVP elution was detected from the non-PSu membrane dialyzer.

Then, we increased the flow rate of the circulating saline to 200 mL/min and determined the time-dependent profiles of PVP *in vitro* elution from each dialyzer. There was no significant difference in the time-dependent PVP elution patterns of each PSu membrane dialyzer between the experiments with a flow rate of 200 mL/min and 100 mL/min (Fig. 3). Furthermore, we found that the increased flow rate did not influence the amount of PVP eluted from each PSu membrane dialyzer at any time point, as shown by the results of the Mann–Whitney *U* test for the two groups of 100 mL/min and 200 mL/min flow rates. In HD, blood flow rates <200 mL/min increased the risk of 1-year mortality [21]. These results suggest that circulating the patient's blood in the circuit at a blood flow rate >200 mL/min would be desirable for HD with PSu membrane dialyzers.

### 3.3 Evaluation of the effect of 24 h incubation with saline without its circulation on PVP *in vitro* elution from the primed dialyzers

**Fig. 4:**
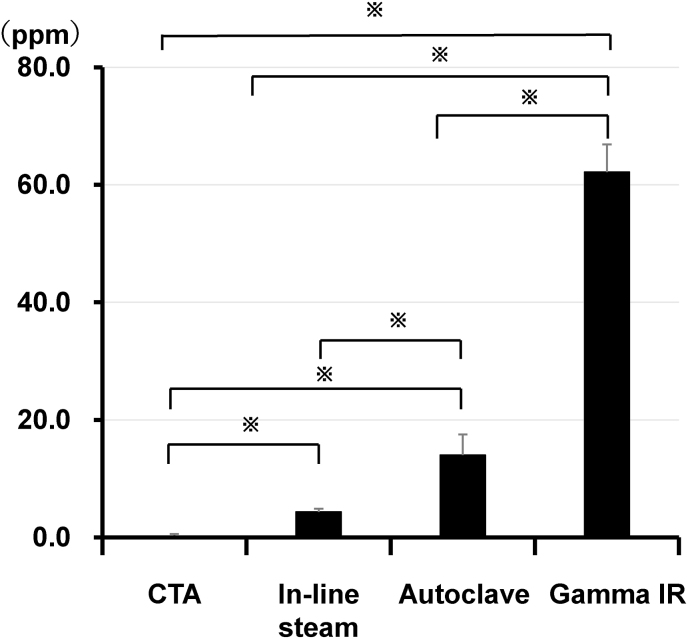
Profiles of *in vitro* polyvinylpyrrolidone elution from the primed dialyzers incubated with saline for 24 h without its circulation. Each primed dialyzer was set in the circuit as described in Fig. 1C and was then incubated with saline at 25 °C for 24 h without its circulation. After incubation, the saline was recovered for the measurement of the polyvinylpyrrolidone (PVP) amount using spectrophotometry. Values represent the mean ± standard error of the six independent experiments. ^**※**^P < 0.05. Abbreviations: Gamma IR; polysulfone dialyzer (Gamma irradiation), Autoclave; polysulfone dialyzer (Autoclave), In-line steam; polysulfone dialyzer (In-line steam), CTA; cellulose triacetate membrane dialyzer.

The fact that PVP was detected when saline was circulated in the circuit connected to the primed dialyzers (Fig. 3) indicated the possibility that incubation of the PSu membranes of the primed dialyzers with saline without its circulation would induce PVP *in vitro* re-elution from them. To test this possibility, we incubated the primed dialyzers with saline for 24 h without its circulation, and recovered the saline to determine its PVP concentration. As shown in Fig. 4, PVP was detected in the saline incubated with any PSu membrane dialyzer. It is noteworthy that the concentrations of PVP eluted from the PSu dialyzer (Gamma IR), PSu dialyzer (Autoclave), and PSu dialyzer (In-line steam) were higher than those detected in the priming (Fig. 2) and the circulation (Fig. 3) experiments with PSu dialyzer (Gamma IR), PSu dialyzer (Autoclave), and PSu dialyzer (In-line steam), respectively (Table 2). These results suggest that the length of time the PSu membrane is immersed in saline is a determinant for the level of PVP elution from PSu membrane dialyzers. In addition, they suggest that non-PSu membrane dialyzers may be appropriate for HD used for CRRT that requires long-term blood circulation [4] because the PVP elution from PSu membranes would reduce the hydraulic permeability of the PSu membrane and decrease the functionality of the PSu membrane dialyze [[7], [8], [9]].

**Table 2:** *In vitro* PVP elution levels in the priming, the circulation, and the immersion experiments corresponding to each of the dialyzers tested.

	Priming with saline (Analytical sample S1)	Saline circulation at a flow rate of 200 mL/min for 8 h	Immersion with saline for 24 h
Cellulose triacetate membrane dialyzer	0 ppm	0 ppm	0.2 ± 0.4 ppm
Polyvinylpyrrolidone (PSu) membrane (In-line stem)	0 ppm	1.5 ± 0.5 ppm	4.3 ± 0.5 ppm
PSu membrane dialyzer (Autoclave)	2.5 ± 0.5 ppm	4.2 ± 0.8 ppm	14.0 ± 3.5 ppm
PSu membrane dialyzer (Gamma IR)	6.5 ± 3.7 ppm	10.5 ± 1.0 ppm	62.2 ± 4.7 ppm

In the co-submitted study [25], when determined by measuring the ultraviolet (UV) absorption at 220 nm of eluted substances, the elution patterns of unidentified substances from the dialyzers tested in the present study were distinct from those of PVP determined by the Müller method, which can specifically detect PVP [26] (Please compare the results in Table 2 of the current study with those in the Table of the co-submitted study). In particular, eluted substances were detected in the non-PSu membrane dialyzer with UV absorption. These results provide evidence that substances other than PVP can be eluted from PSu and non-PSu membrane dialyzers, emphasizing the importance of identifying the eluted substances for safety in HD.

Our results suggest that the circulation duration (time of incubation of the PSu membrane with saline) is a critical factor in determining the PVP elution levels. It should be stressed that the PVP elution levels in the current study were lower than the amount showing toxicity in humans [17], which is evidence of the safety and high quality of the tested PSu membrane dialyzers. However, considering the possibility that PVP elution would reduce the hydraulic permeability of PSu membrane [[7], [8], [9]] as well as the adverse effects induced by long-term accumulation of PVP in the body [[10], [11], [12], [13], [14], [15], [16]], it is important to set up a circulation duration in a dialysis session that is suitable for both, good patient prognosis and reduced PVP elution from the PSu membranes.

### 3.4 Limitation of the present study

It should be stressed that human matrix may have influence on our results from *in vitro* analyses. For example, reports have indicated the formation of a secondary layer membrane due to adsorption of blood proteins in *in vivo* HD where blood is used [27,28]. It is noteworthy that the formation might influence PVP elution from the PSu membrane dialyzers. The current study used saline, an aqueous solution, for the experiments analyzing *in vitro* PVP elution. Thus, future studies with blood perfusion are required to extend our findings to clinical hemodialysis.

We have not provided the data regarding the PVP content in the three investigated PSu membrane dialyzers. Thus, comparing the different amounts of PVP eluates does not enable us to conclude whether different results are achieved because of primarily different PVP content in the membrane material or by the sterilization method in each tested dialyzer brand. Accordingly, the current study compared the *in vitro* PVP elution levels in the priming (Fig. 2), circulation (Fig. 3), and immersion (Fig. 4) experiments corresponding to each tested PSu membrane dialyzer (Table 2) to suggest that the duration of the PSu membrane's incubation in saline is a determinant for the level of PVP elution from the PSu membrane dialyzers.

A part of the eluted PVP would be expected to be eliminated through the hemodialysis treatment owing to the migration of PVP across the dialysis membrane into the dialysis fluid, depending on the molecular weight of the eluted PVP. It should be stressed that our experimental setup in Fig. 4 did not account for the dialytic removal of PVP because we did not circulate saline through the tested dialyzers.

## 4 Conclusions

The current study evaluated the influences of the priming process (washing with saline), dialysis circulation conditions, and saline incubation on PVP elution from three types of PS membrane dialyzers. Notably, when the dialyzers were incubated with saline for 24 h without its circulation, the concentrations of PVP eluted from the three PS membrane dialyzers were higher than the concentrations of PVP detected in the priming and the circulation experiments with the same dialyzers. These results indicated the possibility that the length of time the PS membrane is immersed in saline is the determinant for the level of PVP elution from PS membrane dialyzers. Clinical trials on humans would be important to verify findings from *in vitro* analyses of current study.

## Credit author statement

Y. S. designed, analyzed, and performed the experiments; H. H. performed the experiments; S. F. performed the experiments; S. T. analyzed the data; J. H. analyzed the data and wrote the manuscript.

## Declaration of competing interest

The authors declare no competing financial and non-financial interests.
